# Evaluation of Surveillance Strategies of Antimicrobial Consumption in Animals

**DOI:** 10.3390/antibiotics13060505

**Published:** 2024-05-29

**Authors:** Carly Ching, Muhammad H. Zaman, Veronika J. Wirtz

**Affiliations:** 1 Department of Biomedical Engineering, Boston University, Boston, MA 02215, USA; zaman@bu.edu; 2 Center on Forced Displacement, Boston University, Boston, MA 02215, USA; 3 Department of Global Health, Boston University School of Public Health, Boston, MA 02118, USA; vwirtz@bu.edu

**Keywords:** surveillance, antimicrobial consumption, animal, antimicrobial resistance (AMR), One Health

## Abstract

The aim of this paper is to explore and assess various strategies for monitoring antimicrobial consumption (AMC) in animals, within the context of the One Health approach. Recent studies have shed light on the limited surveillance and data collection for AMC in animals. Using the United States Center for Disease Control and Prevention Policy Analytical Framework, we assess global, national, and farm-level surveillance strategies on public health impact and feasibility using evidence from primary, secondary, and grey literature. From this, we identify key policy mechanisms that support the adoption of surveillance while providing specific recommendations. We find that a global strategy, though valuable for benchmarking and policy guidance, faces participation and data visibility challenges. National-level surveillance offers direct inputs into national action plans but struggles with data uniformity and comparability. Farm-level surveillance, while resource-intensive, provides the most granular data for informing specific interventions. We advocate for a multi-faceted approach to AMC surveillance, emphasizing that legal mandates and financial incentives are crucial for encouraging surveillance participation, along with international cooperation for enhancing participation and data quality. Drawing parallels with public reporting challenges in other sectors can provide valuable lessons on how to address data collection, analysis, and reporting barriers.

## 1. Introduction

Evidence suggests that the indiscriminate veterinary use of antibiotics and antimicrobials in animals may be a critical driver of bacterial antimicrobial resistance (AMR) [1], which renders antimicrobials ineffective. It has been estimated that more antibiotics are consumed by food-producing animals than humans, and high levels of antimicrobial use in animals are concerning, as there are multiple routes for spillover between animals, the environment, and humans (a concept encompassed by the One Health approach) [2]. Maintaining the efficacy of antimicrobials for animal health and welfare is also critical to food security and economic development [3]. To set measurable policy targets and assess the effectiveness of interventions and policies aimed at reducing the indiscriminate use of antimicrobials in animals (and especially the use of antibiotics medically important to humans), reporting and surveillance for estimating antimicrobial consumption (AMC) are required [4]. A lack of reporting challenges the ability to evaluate policy changes on actual use [5]. Identifying different surveillance strategies for antimicrobial consumption in animals and prioritizing solutions that are the most amenable to collecting robust data in low-resource settings are important for policymakers and program implementers. 

There is a growing body of work that reviews and evaluates National Action Plans (NAPs) for AMR. A recent analysis of all available NAPs [6] identified gaps including the lack of legislation in the animal health sector and a lack of integrated AMC and AMR surveillance systems, with a specific lack of data collection for agriculture and animal health. A scoping review on the implementation, monitoring, and evaluation of antimicrobial resistance surveillance systems identified overarching themes (the capacity for surveillance, data infrastructure, policy, representativeness, stakeholder engagement, and sustainability) and found that the literature focuses primarily on human surveillance strategies [7]. While robust surveillance is recognized by international organizations as critical in fighting AMR [3,8,9], differing structures of agricultural and healthcare systems and the heterogeneity of data availability and resources make these activities difficult to standardize. Proposed frameworks for measuring veterinary AMC consumption include evaluating the integration of One Health in surveillance systems [10] and approaches tailored to LMICs [11]. Within the literature, there are overviews and reviews of key components of specific AMC systems [12] or specific surveillance strategies [13]. We, however, found no current comparative policy assessments, to our knowledge, of the different strategies for AMC surveillance in animals. This is an important gap given the diversity of current systems and experiences with surveillance systems [7] and the multiple strategies by which AMC can be monitored. We specifically aim to identify which strategies are most feasible and impactful along with key policy mechanisms that support the adoption of surveillance while providing specific recommendations. 

The goal of antimicrobial consumption (defined as a catch-all term including (combined or stratified) antimicrobial sales and use data [14]) surveillance is to report the number of antimicrobials used (numerator) in the animal population (denominator). This can range from high-level metrics (i.e., total antibiotics used/total animal mass) to more granular data which include specifics on use (i.e., specific antibiotic used for growth promotion/specific animal). For higher-level surveillance, data for the numerator is typically collected through sales or import data of veterinary antimicrobials, while data for the denominator typically uses global or national statistics databases, often using standard animal weights to calculate a population correction unit [15]. Currently, there is no one standardized metric for reporting antimicrobial consumption in animals, and harmonization will be important moving forward [16]. 

Hence, the objective of this paper is to review and assess different surveillance strategies for antimicrobial consumption in the veterinary sector by adapting the United States Center for Disease Control and Prevention (CDC) Policy Analytical Framework [17] using primary, secondary, and grey literature. Specifically, we identify and describe the strategy options, describe each option using key framing questions as a guide [17], and assess the public health impact and feasibility of each strategy. While we touch on the economic impact, due to limited information and varied contextual factors, we did not assess budgetary impacts. After our assessment, we provide a comparative analyses table highlighting the impact, feasibility, and the balance between operational challenges and the potential to inform policy. We continue our analysis to examine and prioritize solutions most amenable to collecting robust data in low- and middle-income countries (LMICs), where there are especially limited surveillance and data about agricultural antimicrobial usage, partnered with an increased risk for AMR due to the increased intensification of farming [11,18,19]. 

## 2. Assessment

Below, we review and assess three major strategies for collecting antimicrobial consumption data in food-producing animals: global-, national-, and farm-level surveillance.

### 2.1. Strategy 1: Global-Level Surveillance 

#### 2.1.1. Overview

Standardized global-level surveillance is a potential strategy. On the global scale, the World Organization of Animal Health (WOAH) performs surveillance among its member countries and certain non-contiguous territories and non-WOAH member countries, recently launching the online Animal Antimicrobial Use (ANIMUSE) global database [20,21,22]. This initiative uses an organizational policy lever and aligns with the WHO 2015 Global Action Plan (GAP) and WOAH Strategy on AMR and Prudent Use of Antimicrobials. Participation is voluntary and involves a template and guidance documents which are provided to national delegates and focal points for data collection [21]. The basis of data collection stems from the WOAH Terrestrial Code Chapter 6.9 on “Monitoring of the Quantities and Usage Patterns of Antimicrobial Agents used in Food-producing animals” [23]. This chapter is part of the WOAH international standards adopted by member states and, while not mandatory, provides some external and peer pressure for adhering to the code. However, in 2012, the WOAH surveyed member states on the implementation of the WOAH Terrestrial Code chapter and found that only 27% of responding members had an official surveillance system. Thus, in 2015, WOAH adopted a resolution mandating WOAH to gather data on the use of antimicrobials in animals, supported by the Quadripartite collaboration on One Health [22]. The initiative is also financially supported by the Fleming Fund. 

For reporting, data are presented as normalized results expressed in milligrams of antimicrobials reported per kilogram of estimated animal biomass. The data mainly come from sales and import figures either at the class or subclass level. Animal biomass is calculated for food-producing species as a total weight using data from the World Animal Health Information System (WAHIS) and the Food and Agriculture Organization of the United Nations Statistical Database (FAOSTAT). The 2023 report (representing the seventh round of data collection for the 2019 calendar year) collected data from 157 countries, with 120 providing quantitative data [22]. However, the data are presented on an aggregated regional level, as making country-level data public is optional, as the WOAH aims to “foster the participation without pointing out potential gaps in national capacities” [24]. Currently (20 February 2024), only 39 national reports are online, with the majority from European countries. Twenty-one countries are opting to make data publicly available in either the eighth or ninth Collection round. Of those 21 countries, 17 are in Europe, 1 is in Africa (Senegal), 1 is in Asia (Sri Lanka), and 2 are in Oceania (New Zealand and New Caledonia) [25]. This suggests a capability but hesitation for public-facing surveillance in many countries. 

#### 2.1.2. Public Health Impact (Strengths and Weaknesses)

Having a central harmonized surveillance system allows for standardized and uniform data collection which reports using the same metric. This can have an impact on the global scale in benchmarking against different countries to determine the effectiveness of global and national strategies for reducing AMC in animals. This can also provide reliable datasets for analyzing against different policies and antibiotic resistance data. Indeed, a major limitation in studying the relationship between different drivers, such as animal AMC and antibiotic resistance, has been data quality and the sparsity of data [26]. This can have a further impact when integrated with human surveillance. However, on the global level, the WOAH does not directly make decisions about national-level policies, although countries are urged to use the data in these contexts. Thus, there may be less of an initiative for the surveillance data to move forward national policies. Policies that stem from surveillance to reduce AMC in animals do have the potential to negatively impact the health of animals and pose a threat to food safety or food security [27]. Since the data are aggregated, there is a lack of a resolution on species and usage, as well as unknowns about data quality. 

#### 2.1.3. Feasibility

On the operational side, major barriers preventing participation in ANIMUSE by countries include a lack of coordination between national ministries of health, a lack of structure or enforcement of a regulatory framework, data quality, and issues with IT systems [22]. Participation assumes that the data exist (records are maintained) and includes time invested to retrieve and input the data. Countries that have these data in robust digitized formats will have an easier time collating the data for input. Countries with legislation requiring data on import, sales, or the use of antimicrobials used for food-producing animals to be reported are in a much better position to collect data. ANIMUSE provides countries with a nine-month period for collecting data for each annual round (between September and May). 

Global surveillance is typically driven by global stakeholders that align with the public health priorities of AMR and One Health. A high participation in ANIMUSE but low participation in making national-level data public suggests hesitation or opposition to public-facing reporting of the data. Historical opponents to regulations involving antibiotics and animals (which may stem from surveillance) include politicians, drug manufacturers, and farmers themselves [28,29,30]. This is often due to the economic and temporal burden associated with new policies, including changing practices to limit antimicrobial use, especially for growth promotion. Farmers will want to adopt practices only if their benefit exceeds the costs. Thus, if the cost is higher, incentives may be required [31]. Another concern is disbelief in the underlying scientific evidence, or a lack of knowledge. Specific to the release of AMC data, concerns include privacy, autonomy, judgement, and mischaracterization of the data [32,33]. To counter security concerns, ANIMUSE ensures a robust authentication process, encrypts data, and actively monitors potential threats [24]. 

Expanding on the economic consequences, the pharmaceutical industry, pharmacies, and veterinarians could lose money if livestock workers use less antibiotics. It has been estimated that the total global market value of farmed animals is between USD 1.61 and 3.3 trillion (2018) [34]. The economic impact and burden on animal health because of reducing antimicrobial use in livestock production is a major argument against policies. There is currently varied evidence on the impact, which is context-dependent [27]. Subsidizing or providing other incentives for reporting data may be another strategy when data reporting is not mandatory or does not have a legal basis [31]. 

### 2.2. Strategy 2: Regional/National-Level Surveillance (Using Sales Data)

#### 2.2.1. Overview

Another strategy is regional and national-level surveillance programs that provide country-level data. These typically use higher-level sales data or import data from various sources including market authorization holders, wholesalers, manufacturers, importers, feed mills, retailers, veterinarians, farmers, and compounders for the numerator. Most active and public national-level surveillance networks for AMC in animals that exist are primarily in high-income countries [35], although surveillance is a target activity for many National Action Plans [36], with an increased number of LMICs reporting established or starting the implementation of integrated surveillance [37]. These typically use legislative and regulatory levers. The European Surveillance of Veterinary Antimicrobial Consumption (ESVAC) [38] is an example of a voluntary surveillance system, which has been a precursor to legislation requiring EU/EEA member states to report data on volume or sales and the use of antimicrobial medicinal products in animals, starting in January 2024 [39]. The ESVAC program began in 2008 with a request from the European Commission to the European Medicines Agency to lead an effort to collate data collected by member states on the use of antimicrobial agents in animals. ESVAC was voluntary but had a clear political mandate, bolstered by a sufficient legal basis to request the pharmaceutical industry provide sales data to national authorities. The legal basis includes laws mandating the collection of sales data [38]. This underlies the data availability that allows for data reporting, collation, and the calculation of consumption metrics. With the implementation of ESVAC, the EU was able to measure a decrease of 53% in aggregated sales of antibiotic veterinary medical products between 2011 and 2022 for 25 countries [38]. This reflects the introduction of antimicrobial stewardship efforts and legislative restrictions [40,41].

Indeed, many countries with active surveillance programs began with the voluntary collection/reporting of data but moved to regulatory changes requiring the reporting of sales or import data [14]. Thus, surveillance systems are more sustainable when there is a legal basis for the collection of sales or usage data but may require initial voluntary pilot phases for the collection and collation of data by intermediate parties. In these systems, data providers upload their data to the electronic database using standardized forms [15,38] each calendar year. Metrics are then calculated using a defined methodology. Some strategies attempt to stratify sales data by species.

There remains a significant gap in the surveillance of antibiotics in animals in LMICs, though a framework for surveillance suggests a phased approach [11]. Thailand is one of the first middle-income countries to implement a national-level surveillance system for antibiotic consumption in food-producing animals [12,42,43,44], which is supported by the World Health Organization Country Cooperation Strategy (WHO-CCS), a multi-funding platform including governmental, national, and international agencies. Consumption is estimated based on volumes of registered products or feed and medicated feed produced or imported (minus the quantity of exports), which is mandated to be reported to the FDA by the Drug Act or Animal Feed Quality Control Act. As these data are already mandatory, electronic records can be retrieved and analyzed using a set methodology. 

#### 2.2.2. Public Health Impact

Like global surveillance systems, national-level surveillance can further public health objectives that require determining and monitoring AMC in animals. Robust systems for collecting data on usage in animals have been more successful in implementing policies than those that lack these systems [45]. A benefit of national-level systems is that the data can feed directly into National Action Plans and allow for the monitoring of country-specific policies. Since the country is more involved, more political changes may be made if it is part of a broader framework and public health goal. However, data collected by national or regional surveillance systems may not be uniform or comparable to other countries when the metrics are not uniform (i.e., mg/PCU vs. mg/kg). Additionally, there may not be a secondary quality check performed by an outside party. Another limitation is that sales or import data are not the same as usage data, and a lack of a context may lead to the overinterpretation of the data or hide gaps and weaknesses in current policies on a more granular level. Surveillance based on sales data may not be representative of true AMC at the farm-level, especially in countries with high levels of off-label use, higher waste (the sold antimicrobial volume that is never consumed), or easier access to antimicrobials without a prescription. 

#### 2.2.3. Feasibility

National or regional surveillance systems require coordinated support between different stakeholders, including governments and end-users, political prioritization, and economic investment. The current landscape of active public systems suggest that it is more feasible when the surveillance or data reporting is legislated (even if the original law was not intended for specific AMC surveillance initiatives). Operationally, surveillance systems require the development of a specific protocol and automated systems and IT networks for data collection and collation. ESVAC started in 2009 with nine countries and took until 2022 to report sales in 31 countries (reporting will now be mandated following new legislation taking effect [41]), and it has yet to sustainably report farm-level use data (expanded on below) [38]. 

There are often political barriers to passing laws to collect data or report data. For example, the United States has failed to pass legislation for further disclosure about antibiotic use in animals multiple times [28]. The emphasis on urgency is often varied by authoritative figures, leading to conflicting views on policies on AMC and animals. Politicians are likely to not support surveillance initiatives and legislation if it will lose them political support or campaign donations by their constituents, among which industry figures, livestock workers, and pharmaceutical companies are often major interest groups. Again, this lack of support for reporting data often stems from not wanting restrictions on antimicrobial use in animals, for which opponents fear the data would be used [28]. Similar to the above, some of the biggest barriers are the perceived risks to costs, profits, food security, and animal health, along with the extra work involved [6]. 

### 2.3. Strategy 3: Farm-Level Surveillance 

#### 2.3.1. Overview

In the absence of national surveillance using high-level sales data, farm-level surveillance for antimicrobial consumption in food-producing animals is another strategy. Of note, this can also be a complementary strategy for collecting more detailed usage data when there is national-level surveillance. A major advantage of farm-level surveillance is that it can address some of the limitations described above in that it captures off-label use and provides more details about species-specific usage to inform specific interventions. A recent review from 2020 [13] provides an overview of current farm-level systems and their components, which were all located in Europe, except for one in Canada. This review was written within the framework of the AACTING project (a global “network on quantification of Antimicrobial consumption in animals at farm level and Analysis, CommunicaTion and benchmarking to improve use”), which has also published guidelines for collecting data on the farm level [46] and an overview of current systems, including a methodology and legal basis [47]. 

The ESVAC program also performed a pilot for collecting farm-level data in 2014, which noted operational barriers. However, the European Union is now requiring usage data to be reported for certain animals in collection year 2024 in accordance with the published guidance for minimum data requirements that arose from the pilot project [48]. In the ESVAC trial, ten volunteer member states collected data on antimicrobial usage in pigs using on-site visits of a small convenience sample of five farms for one year using two templates [49]. 

In the United States, there is an ongoing project developing a framework for a public–private partnership for collecting data on antimicrobial use in animals [32,50]. Various methods for collecting data are presented in academic studies. These include farm-level surveys/questionnaires [51,52], farm and veterinary records [53,54] and garbage can audits [53,55], which is a methodology in which all packaging is collected and audited. Studies that tested the agreement between methods show a lack of consistency between data sources [53,54], but the methods involving software are better than manual collection [54], and one found almost perfect agreement between a garbage can audit and data collected from veterinary invoices [53]. 

#### 2.3.2. Public Health Impact

Farm-level surveillance provides the most data about usage, including off-label use, which can inform more specific interventions (e.g., at the species level) and may provide more accurate data. It can also help build farmer–veterinarian relationships and trust through longitudinal partnerships. Indeed, the collection of antimicrobial use data, including the context for use including the treatment regime, disease indication, and animal size, provides a more accurate picture for optimizing a stewardship and evaluating associations between antimicrobial use and AMR [32,50].

#### 2.3.3. Feasibility 

The ESVAC pilot on pig farms conducted a feasibility survey of the representatives of the volunteer countries [49]. Barriers included the difficulty convincing farmers to participate and the time needed to fill out the templates. The survey also found that an automated continuous data collection system made it possible to extract most of the required data from a database, and using health records and logbooks as a data source appeared to take more time than using prescriptions/practice records. 

After the pilot, ESVAC found [56] that there was an insufficient level of support for conducting an expanded study due to issues such as the cost of on-farm surveys, the lack of a legal basis, the complexity of the project planning process, and demanding resources for “manual” collection, suggesting it would not be a sustainable approach [56]. Instead, recommendations arose for national automated or semi-automated data collection from all farms or a representative sample of farms. ESVAC has produced guidance for the collection of harmonized and standardized use data, which outlines the minimum required variables, along with a template that countries can populate using data collected for this purpose or as part of (existing) national surveillance schemes [48]. Common themes from the roundtable and public meeting discussion among stakeholders exploring a public–private partnership for collecting data on antimicrobial use in animals in the US included challenges collecting standardized data across species and routes of administration and suggested that encouragement through incentivizing through benchmarking methods or monetary methods may be required, especially for a voluntary system [50]. Another concern is trust surrounding data access and privacy. For example, farmers may have hesitations if a third party is collecting data on site and fear consequences for the indiscriminate or off-label use of antibiotics. Accordingly, these data would have to be reported in an anonymized manner or aggregated such that individual farms or prescribers would not be identifiable [48].

Overall, farm-level surveillance is resource-intensive, onerous, and disruptive to the farmer and veterinarian, depending on involvement. It is also harder to validate data quality, including inconsistent or missing data, depending on who is reporting the data and how it is being collected [50]. Given the potential misrepresentation of data, there should be defined guidelines on how data are used. Another gap is understanding the explicit level of involvement of farmers/end-users in the development of methodology compared that of industry stakeholders or trade organizations, policymakers, and experts on collecting AMC data [50]. This added agency can identify practical gaps. However, in the absence of aggregated national data, farm-level data collection is a valuable alternative.

Table 1 summarizes each of the surveillance levels, including the balance between operational challenges and the potential to inform policy.

## 3. Discussion

From the review and analyses of the three strategies presented above, we identify an imbalance between feasibility and impact among surveillance using sales data and farm-level surveillance networks. Ideally, sales and usage data would be integrated at any level and a strategic combination of approaches could triangulate the issues of universal coverage, understanding farm-level use and practices, and estimating under-reporting and misuse. However, if a country only has the resources for either Strategy 2 (Regional/National-level sales surveillance) or Strategy 3 (Farm-level surveillance), high participation in the ongoing WOAH ANIMUSE database (157 countries) suggests that it will be more feasible to report basic metrics of sales data along with the total biomass taken from national statistics. Sales data may also have less reporting bias than self-reported data; however, they may not accurately capture use. Questions about data quality remain for the underlying data of aggregate reporting, however. Governments opting for enacting a legal basis for collecting sales data from producers and sellers can achieve a more rapid rollout of national-level surveillance. 

Given the ability of ANIMUSE to identify trends over multiple years based on the normalized number of milligrams of antimicrobials used per kilogram of estimated animal biomass [22], the phased approach for LMICs seems appropriate [11]. Specific to LMIC contexts, there are gaps in understanding the feasibility in low-resource settings, where the economics of implementing farm-level surveillance may be different. For example, clinical trials in LMICs tend to cost less than trials in HIC, attributable to the lower salaries of healthcare workers and study coordinators [57]. In countries lacking national-level surveillance data and which resist laws that mandate the reporting of antimicrobial sales, farm-level surveillance is a critical alternative. Standardization to ensure data quality and comparability is needed alongside, investments in data collection and human capacity. Legislation can drive data capture; however, enforcement will also be critical. Indeed, when antibiotic stewardship regulations are introduced without proper enforcement, changes in AMC are not observed [58]. There may also be less of an incentive to circumvent laws requiring the collection of sales data due to fewer perceived negative consequences that can arise from bulk volume data as opposed to information about farmers using antibiotics indiscriminately. 

Voluntary schemes for reporting AMC without a legal requirement may have to utilize subsidies or incentives, which programs promoting the transition from conventional to organic farming have successful employed. Organic farming standards ban the use of all antibiotics, which requires the verification of AMC. As such, lessons can be learned from policies for organic farming, which also contribute to the reduction in the use of antimicrobials. A review of found that the barriers to adopting organic farming included attitudes (being risk-averse) and beliefs (believing it to be unsuitable and complicated in practice). Other barriers were climatic conditions, the abundance of pests, certification systems, and marketing problems. Supportive factors included “training, extension agency communication, information acquisition, membership of an association, access to resources and markets, technology support, motivation/subsidy and organic farmer neighbors, and others”. Motivation programs from the government, non-government, and business sectors also had a positive impact [59]. 

Subsidies are currently paid to farmers in many countries to maintain minimum levels of production and maintain the standard of living. Further subsidies for organic farming serve as an effective direct supply-side policy instrument that some countries utilize. Denmark, which has the highest organic market share, has been a leader in organic farming policy [60]. It was the first country to enact a distinct organic farming law in 1987 and introduced subsidies for the conversion from conventional to organic farming, followed by permanent organic farming subsidies in 1994. However, in 2004, permanent organic subsidies ended, and farmers were paid an environmental subsidy, with organic farming given priority [61]. In the UK, the Organic Farming Scheme (OFS) included higher organic farming support payments. The OFS led to an increase of 150,000 ha of organically managed land in nine months, and the scheme was forced to close after six months due to the allocated budget being used up. In June 2003, on-going support after conversion was introduced for a five-year period. The OFS closed to new entrants in March 2005 and was replaced by the Organic Entry Level Stewardship, with organic farmers receiving twice the conventional rate, along with conversion payments [61]. Thus, subsidies are an effective tool for conversion and maintaining alternative farming practices but may run into budget constraints and need to be modified along the way. In Europe, consumer sentiments have turned towards farming with lower levels of AMC. Export opportunities of farmers from other countries into Europe or other geographies with stricter limits on antimicrobials could incentivize large, export-producing farming businesses to report and adhere to regulations. 

The issues and concerns voiced for feasibility, including the time, costs, poor data quality, and risk of data misinterpretation, are not unique to reporting AMC in animals. These same concerns are commonly voiced about public reporting in other fields. For instance, opponents to public reporting about the quality and costs of healthcare systems argue that costs are high and also highlight how electronic record-keeping systems facilitate lower-cost reporting, that the information collected is not accurate or reliable, and that there is a risk of misinterpretation by the public lacking field-specific literacy [62]. Opponents to the reporting of prices by health systems argue that reporting takes too much time and is not a priority compared to other initiatives [63]. Laws for reporting have stemmed from specific historical events. For example, the US Securities Act of 1933 for regulating the offer and sale of securities, by which all public companies must register with the U.S. Securities and Exchange Commission (SEC) and disclose specific business and financial information, arose from the stock market crash of 1929 and the Great Depression [64]. However, data granularity and detail remain a common issue [65]. And even though there is pushback for hospital reporting, participation is incentivized and penalized, and these incentives make the voluntary system almost a requirement due to the financial loss associated with not participating [66]. The pay-for-reporting program of the Centers for Medicare & Medicaid Services (CMS) Hospital Inpatient Quality Program allows CMS to pay hospitals that successfully report designated quality measures a higher annual update to their payment rates, with a reduction in one-quarter of the payment rate update if all requirements are not met. Data from selected measures are also used under value-based purchasing programs that reward providers for the quality of care they provide [67]. Thus, laws (mandatory) or financial incentives/penalties are two major effective policy mechanisms for public reporting. 

Within the One Health approach, integrated AMC surveillance includes data not only for animals but also for humans and the environment [8,9]. This would provide the most complete picture of AMC required to inform policy and to evaluate the effectiveness of interventions and policies on multiples levels. This would require multi-sector collaboration and could be supported by establishing collective action groups with stakeholders from all relevant professional groups. 

## 4. Conclusions

There are multiple strategies for the surveillance of AMC in animals that can provide relevant data to inform and monitor policy, with existing frameworks to build upon. We find that governments that rely on a legal basis for collecting sales data from producers and sellers can achieve a more rapid rollout of national-level surveillance. In addition to legislation, financial incentives and penalties are effective policy mechanisms that will enable participation in surveillance schemes for AMC in animals. We identify that AMC data likely exist but will require further work to promote data sharing and to evaluate data quality. Strategies and supportive factors that promote the adoption of AMR and animal stewardship policies and public reporting can be informed by other sectors and alternative agricultural systems.

## 5. Recommendations

From our review, we identify the following key research questions and recommendations:

1. How can countries be convinced to prioritize public Animal–AMC surveillance and reporting within AMR National Action Plans or pass legislation to make data public and accessible?
2. To assess the validity of pushback due to economic consequences, evaluate what the cost of implementing surveillance is compared to the short-term and long-term economic consequences (including money saved by preventing resistance infections (humans and animals) and the loss of livestock). Evaluate the economics of incentives (or penalties) as a tool for participation.
3. Evaluate the human effort and time needed to implement data collection and surveillance for national-level surveillance and farm-level surveillance. This can help determine if a voluntary activity is fair or sustainable and inform time allocation or fair compensation.
4. To better understand differences in support and acceptability, examine the effective framing of campaigns and policies on antimicrobial use in animals. For example, a recent analysis found differences in the framing of AMR as an economic threat in international documents but as a human development and equity issue in Pakistan [68].
5. Involve farmers/end-users in the development of methodology in collecting usage data. This added agency can identify practical gaps and inform practical guidelines.
6. Develop standardized guidelines on how data are used and urge policies for championing that data are not overinterpreted.
7. Design easily implemented automated systems for data collection and collation and encourage sharing between countries of automated system frameworks and software.

## Figures and Tables

**Table 1:** Overview of the three levels of surveillance using the CDC Policy Analytical Framework.

	Public Health Impact	Feasibility	Balance between Operational Challenges and Potential to Inform Policy
Global surveillance	Provides standardized, harmonized data for global benchmarking and analysis. Can impact global and national strategies for reducing AMC.	Currently voluntary participation in WOAH ANIMUSE; challenges include a lack of coordination, the regulatory framework, and IT systems issues. High participation but low transparency due to confidentiality.	High operational challenges due to voluntary participation and coordination issues, but it has a high potential for informing global policies and interventions due to standardized data collection and reporting.
National surveillance	Allows for the monitoring of country-specific policies and directly feeds into National Action Plans. Can be more successful in implementing policies with robust data systems.	Requires coordinated support, political prioritization, and economic investment. Feasibility increases with legislative backing for data collection.	Moderate operational challenges, with a need for political and economic investment, but a high potential for informing national policies and interventions with country-specific data.
Farm-level surveillance	Provides granular data on usage, including off-label use, informing more specific interventions. Builds farmer–veterinarian trust and can optimize stewardship and evaluate AMR associations.	Resource-intensive, with operational barriers such as data privacy concerns and convincing farmers to participate. Feasibility varies widely with local contexts, being particularly challenging in low-resource settings.	High operational challenges due to resource demands and privacy concerns, but a very high potential for informing specific interventions and policies with detailed and accurate usage data.

## Data Availability

No new data were created.
